# Retraction: The design of tourism product CAD three-dimensional modeling system using VR technology

**DOI:** 10.1371/journal.pone.0305242

**Published:** 2024-06-06

**Authors:** The PLOS ONE Editors

The *PLOS ONE* Editors retract this article [1] because it was identified as one of a series of submissions for which we have concerns about peer review integrity and similarities across articles. These concerns call into question the validity and provenance of the reported results. We regret that the issues were not identified prior to the article’s publication.

YD agreed with the retraction. SYH, JL, JR, WF, and TS either did not respond directly or could not be reached.
